# Transglutaminases and Obesity in Humans: Association of *F13A1* to Adipocyte Hypertrophy and Adipose Tissue Immune Response

**DOI:** 10.3390/ijms21218289

**Published:** 2020-11-05

**Authors:** Mari T. Kaartinen, Mansi Arora, Sini Heinonen, Aila Rissanen, Jaakko Kaprio, Kirsi H. Pietiläinen

**Affiliations:** 1 Faculty of Medicine (Experimental Medicine), McGill University, Montreal, QC H3A 0J7, Canada; mansi.arora@mail.mcgill.ca; 2 Faculty of Dentistry (Biomedical Sciences), McGill University, Montreal, QC H3A 0J7, Canada; 3 Obesity Research Unit, Research Program for Clinical and Molecular Metabolism, Faculty of Medicine, University of Helsinki, 00014 Helsinki, Finland; sini.heinonen@helsinki.fi (S.H.); Aila.Rissanen@medi.inet.fi (A.R.); kirsi.pietilainen@helsinki.fi (K.H.P.); 4 Department of Public Health, University of Helsinki, 00100 Helsinki, Finland; Jaakko.Kaprio@helsinki.fi; 5 Abdominal Center, Obesity Center, Endocrinology, University of Helsinki and Helsinki University Central Hospital, 00014 Helsinki, Finland

**Keywords:** Factor XIII-A, *F131A*, obesity, weight gain, adipocyte size, inflammation

## Abstract

Transglutaminases TG2 and FXIII-A have recently been linked to adipose tissue biology and obesity, however, human studies for TG family members in adipocytes have not been conducted. In this study, we investigated the association of *TGM* family members to acquired weight gain in a rare set of monozygotic (MZ) twins discordant for body weight, i.e., heavy–lean twin pairs. We report that *F13A1* is the only *TGM* family member showing significantly altered, higher expression in adipose tissue of the heavier twin. Our previous work linked adipocyte *F13A1* to increased weight, body fat mass, adipocyte size, and pro-inflammatory pathways. Here, we explored further the link of *F13A1* to adipocyte size in the MZ twins via a previously conducted TWA study that was further mined for genes that specifically associate to hypertrophic adipocytes. We report that differential expression of *F13A1* (ΔHeavy–Lean) associated with 47 genes which were linked via gene enrichment analysis to immune response, leucocyte and neutrophil activation, as well as cytokine response and signaling. Our work brings further support to the role of *F13A1* in the human adipose tissue pathology, suggesting a role in the cascade that links hypertrophic adipocytes with inflammation.

## 1. Introduction

Transglutaminases (protein-glutamine γ-glutamyltransferase, TGase, TG, EC 2.3.2.13) are a family of enzymes that catalyze a Ca^2+^- and thiol-dependent posttranslational modification of glutamine residues [1,2,3,4]. These reactions include acyl transfer reaction with primary amines, glutamine deamination if the second substrate is water, and transamidation, i.e., isopeptide crosslinking, if the second substrate is a lysine of a protein polypeptide chain. The isopeptide formation between glutamine and lysine residues, which is by far the most studied reaction of TGs [1,2,3,4], stabilizes proteins and induces formation of protein networks and alterations in substrate protein biochemistry, stiffness, solubility, and cell adhesive features [2,4,5,6,7]. TGs also have cell signaling functions that do not require crosslinking activity [8].

The TG family consists of nine gene products: TG1–TG7 and Factor XIII-A [1,2,4] (Genes: *TGM* (human), *Tgm* (mouse)). Transglutaminases TG2 and FXIII-A have been studied widely in the context of connective tissue hemostasis and differentiation of their resident cell types. While much focus has been on cartilage and bone [9,10,11,12,13,14,15], new research also shows their involvement in adipose tissue. White adipose tissue (WAT) is a form of connective tissue capable of thermoregulation and energy storage and it plays a central role in maintenance of balanced energy metabolism [16]. In caloric excess, WAT expands via preadipocyte proliferation (hyperplasia) and by their differentiation to lipid storing adipocytes followed by their growth (hypertrophy) [16]. The current understanding is that adipocyte size is associated with metabolic consequences of obesity in a manner where smaller adipocyte phenotype predicts healthier outcome, and larger adipocytes are linked to insulin resistance and other comorbidities of excess weight [17,18]. It is not fully understood why the presence of larger adipocytes presents a worse metabolic outcome in obesity; however, this may at least not link to adipocyte mitochondrial dysfunction [19] but rather on hypertrophic adipocyte-mediated initiation of innate immune reaction [20,21,22]. In obesity, prolonged inflammation compromises lipid storage and healthy WAT function, which lead to lipid accumulation to non-metabolic tissues [16,23,24]. This is linked to the development of insulin resistance and T2D [23].

TG2, which is a ubiquitously expressed TG with a plethora of functions in health and disease, has been linked to adipogenesis and obesity by us [25] and by others [26]. We demonstrated that the absence of TG2 in mouse embryonic fibroblasts increased adipogenesis via several pathways, which included decreased Pref-1 production, canonical Wnt/β-catenin signaling, and increased Akt phosphorylation [25]. The research group of Szondy showed that *Tgm2*-deficient mice do not gain more weight compared to their controls, however they exhibit increased inflammation of adipose tissue.

A recent genome-wide association study by Pietiläinen’s group linked FXIII-A to obesity in weight discordant monozygotic (MZ) twins, and further studies showed that seven small nucleotide polymorphisms (SNPs) in the *F13A1* gene are associated with body mass index (BMI) [27]. The work by Kaartinen’s group in turn examined the role and mechanisms of action of FXIII-A in 3T3-L1 adipocytes and investigated the metabolic phenotype of high-fat diet-fed *F13a1*-/- mice. We reported that in cell cultures, the FXIII-A enzyme can act as a switch to modulate insulin signaling from pro-proliferative to pro-differentiating function via regulating plasma fibronectin accumulation into extracellular fibrils [28]. We also showed that *F13a1* deficiency in mice alters the adipose tissue cellularity and increases small and large adipocytes but decreases macrophage infiltration and improves insulin sensitivity of obese adipose tissue [29], suggesting that FXIII-A reacts to weight gain and may have an adverse effect on adipose tissue health. In our most recent work, we examined the association of *F13A1* to human obesity in weight discordant MZ twin pairs, where the association of *F13A1* to human obesity was originally discovered [27]. In this clinical study, we reported that the expression of *F13A1 is* significantly increased in the adipose tissue and adipocyte-enriched fraction of the heavier co-twin, and its differential expression (ΔHeavy–Lean) shows a strong, significant linear correlation with increase in weight, fat mass, and adipocyte volume, and a negative correlation with circulating adiponectin [30]. A further transcriptome-wide association study (TWAS) between Heavy–Lean Δ*F13A1* and changes in full adipocyte transcriptome between the co-twins identified 182 highly *F13A1* correlating transcripts, for which gene enrichment analysis linked cell stress, inflammation, extracellular matrix remodeling, and angiogenesis of the adipose tissue.

The aim of this study was first to investigate the expression of the whole *TGM* family in acquired excess weight in the MZ twins, and secondly to elucidate details of the links of *F13A1* to pathways associated with adipocyte size that may be relevant to metabolism. We report that *F13A1* is the only *TGM* family gene transcript that is significantly linked to acquired weight gain, showing an increase in the heavier twin. *F13A1* associated with 47 gene transcripts that also showed a link to large adipocyte size, which mostly represented genes of neutrophil attraction, inflammasome, and secretion of pro-inflammatory cytokines. Our study brings further evidence to the relevance of FXIII-A/*F13A1* in adipose tissue health.

## 2. Results

**Figure 1:**
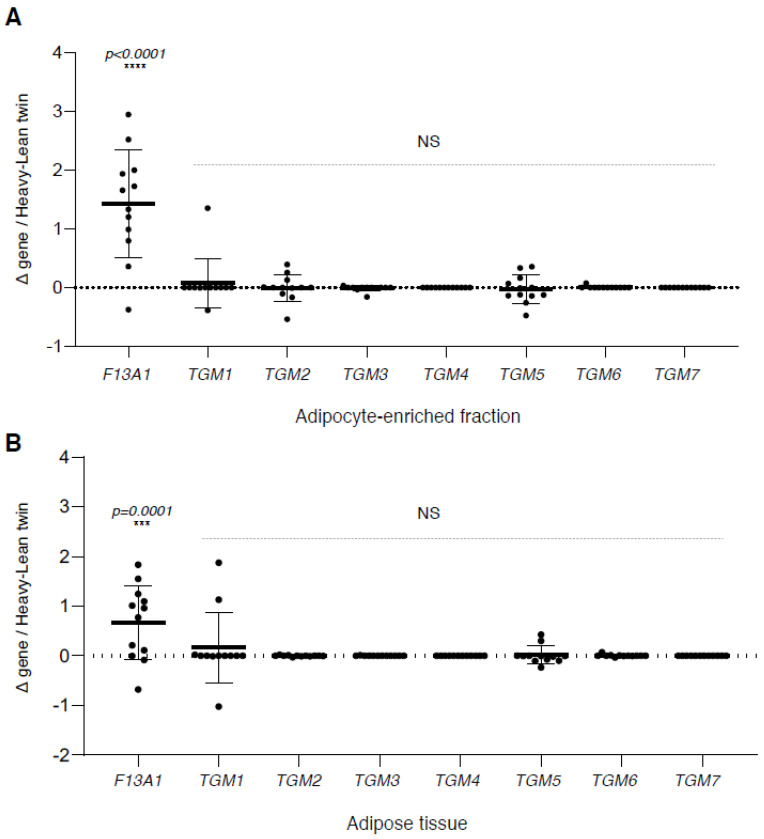
Gene expression of transglutaminase family genes and association of *F13A1* mRNA expression in acquired excess weight. Increase in transglutaminase family (TGM) members in excess weight represented by expression difference (Δ) between heavy and lean monozygotic (MZ) weight discordant twins (*n* = 12 twin pairs). (**A**) Differential expression in adipocyte-enriched fraction. (**B**) Differential expression in adipose tissue. Analysis of variance (ANOVA) with Bonferroni post-test shows that *F13A1* is the sole *TGM* family member that reacts to acquired excess weight in both preparations. No significant changes were observed in other TGM family members. NS, not significant.

Our first aim was to examine potential links of *TGM* family members to excess acquired weight in the 12 weight discordant MZ twins. The significant weight and adipocity discordance of the twins are presented in Table 1. Differential (ΔHeavy–Lean twin) gene expression in adipose tissue and adipocyte-enriched fraction is reflective of cellular changes in the tissue upon its expansion during weight gain. As presented in Figure 1A,B, *F13A1* is the only *TGM* family gene that reacts to excess weight in a significant manner and shows increased expression in both adipose tissue and adipocyte-enriched fraction of the heavier twin [30].

**Figure 2:**
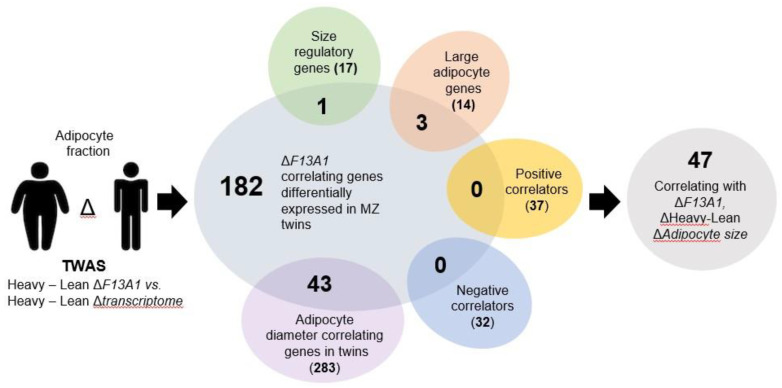
Transcriptome-wide association study (TWAS) strategy to investigate the association of *F13A1* with acquired excess weight and adipocyte size in weight-discordant monozygotic twins. To examine what weight gain and adipocyte size-associated pathways *F13A1* is linked to, we performed a transcriptome-wide association study (TWAS) of differentially expressed and correlated genes (Heavy–Lean Δ*F13A1* vs. Heavy–Lean Δ*transcriptome*). The initial screen identified 182 genes whose differential expression showed significant linear correlation with *F13A1* (r^2^ > 0.5 and *p* < 0.05) and significant differential expression between the Heavy and Lean co-twins (*p* < 0.05) [32]. This set was screened for previously published adipocyte size correlating (positive and negative) genes in the MZ twins, some of which were shared with this study. This resulted in no additional genes. The set was then screened for 14 reported ‘large adipocyte genes’ and 17 genes from the literature that have been published to regulate adipocyte size (PubMed search (‘adipocyte size’ and ‘regulation’ and ‘gene’)). All screened genes are listed in Supplemental Tables S1–S4. The resulting final set of 47 genes that correlated with *F13A1,* adipocyte diameter, and were differentially expressed in the co-twin pairs are listed in Table 2.

Our previous work linked increase in *F13A1* expression to increase in adipocyte size/volume [30]. This, with previous mouse data, suggests that *F13A1* may be increasingly produced by larger adipocytes or may regulate the cell size. In the same study, we identified 182 *F13A1-* and weight-associated genes which showed significant (*p* < 0.05) and strong Pearson correlation (r > 0.7) for Heavy–Lean Δ*F13A1* [30]. Gene enrichment analysis showed that the 182 genes linked to cell stress, inflammation, tissue remodeling, hemostasis, and angiogenesis [30]. To further examine the link of *F13A1* to adipocyte size, we applied the following strategy (Figure 2). First, the pool of 182 genes was further screened for genes that also showed correlation to Heavy–Lean ΔAdipocyte Diameter in the 12 MZ twin pairs. This resulted in a total of 283 genes from which 42 were found as shared with the original 182 gene set. Second, we screened the set for adipocyte size correlating (positive and negative) genes that were identified from 15 MZ twins, some of which were shared with this study [17]. This resulted in no additional genes (Supplemental Tables S1 and S2). Third, we screened 14 reported ‘large adipocyte genes’ from a study where adipocytes, isolated from human SAT, were separated via 70 μm mesh into small (57.8 ± 0.23 μm) and large (95 ± 0.38 μm) adipocytes, followed by mRNA expression analysis and identification of genes that were expressed more than four-fold in large adipocytes versus small ones [18,31] (Supplemental Table S3). From this set, three additional *F13A1-* and weight-correlated genes were identified (*SELE, IL8,* and *LPCAT1*). Fourth, we searched the literature for genes that have been published to regulate adipocyte size. A literature search (‘adipocyte size’ and ‘regulation’ and ‘gene’) resulted in 835 papers, from which 17 reported on genes with strong links to regulation of adipocyte size (associations were not included) (Supplemental Table S4). Only one out of these genes was found in the set of 182 genes (*REPIN1*). Thus, a total of 383 ‘adipocyte size’ genes were screened, which resulted in a final set of 47 gene transcripts that showed significant correlation to Δ*F13A1* and were differentially expressed in the Heavy vs. Lean twin. Classical regulators of adipogenesis and lipid storage, *PPARG, CEBPA, CEBPB,* and *PLIN1,* did not show correlation. The set of 47 genes are listed in Table 2. From this set, 12 genes showed strong negative correlation with ΔWeight-, Δ*F13A1-,* and ΔAdipocyte Diameter. The remaining 35 genes were positive correlators. 

**Figure 3:**
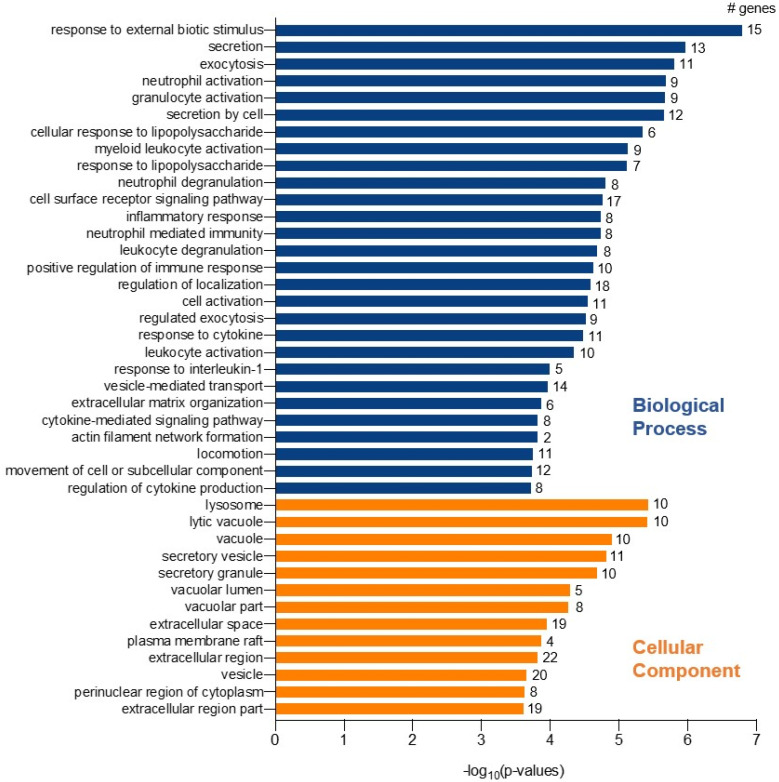
Gene enrichment analysis. Selected over-represented GOterms among the 47 genes whose differential expression correlated with *F13A1*, adipocyte diameter, and showed significantly altered expression between heavy and lean co-twins. Gene ontology analysis was done using GOnet and confirmed with Panther. A total of 61 GOterms were found for Biological Process, and 28 highly relevant are represented here. All 13 Cellular Component terms are shown. No terms for Molecular Functions arose in the search. Significance of the terms is represented as –log_10_ (*p*-value) and number of genes in each GOterm is indicated with the bar. Full set of the GOterms with *p*-values, as well as false discovery rate adjusted *p*-values and list of genes can be found in Supplemental Tables S5 and S6.

**Figure 4:**
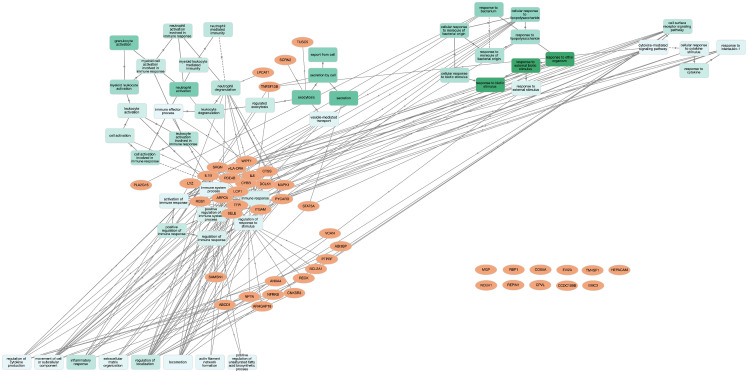
Network of gene ontology terms from 47 genes correlating with *F13A1*, adipocyte size, and acquired excess weight links *F13A1* to inflammatory status of adipose tissue. Gene ontology term, gene clustering, and mapping analysis was performed using GOnet web-application (http://tools.dice-database.org/GOnet/). Analysis is presented as modified Euler layout which reveals three clear functional clusters linked to inflammatory response. The darker color of the GOterm node represents higher significance. The full set of values and GOterms are presented in Supplemental Tables S5 and S6.

**Figure 5:**
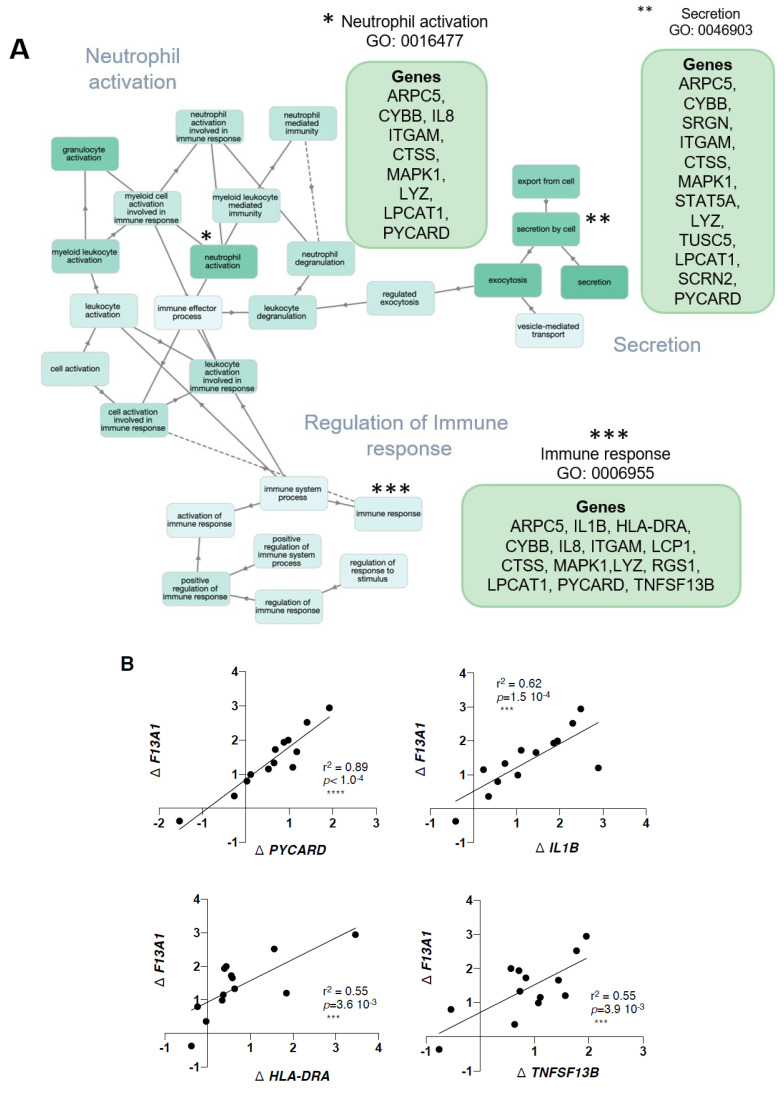
*F13A1* expression in adipocyte-enriched fraction associates with neutrophil activation and early immune response. (**A**) Map of GOterm nodes representing Biological Processes involved in immune response. Node clusters for neutrophil activation, secretion, and regulation of immune response are evident. Gene lists for representative nodes are shown. (**B**) Linear correlation of Δ*F13A1* (Heavy–Lean twin) with differential expression Δ*gene* (Heavy–Lean twin) of selected genes from GOterms. *F13A1* levels increase in adipocyte-enriched fraction together with *PYCARD* (an inflammasome and caspase-1 activator), *IL1B* (interleukin 1b) (early pro-inflammatory cytokine), and *HLA-DRA* (HLA class II histocompatibility antigen presenting factor, and *TNFSF13B* (TNF Superfamily Member 13b and B-cell activating factor)).

**Figure 6:**
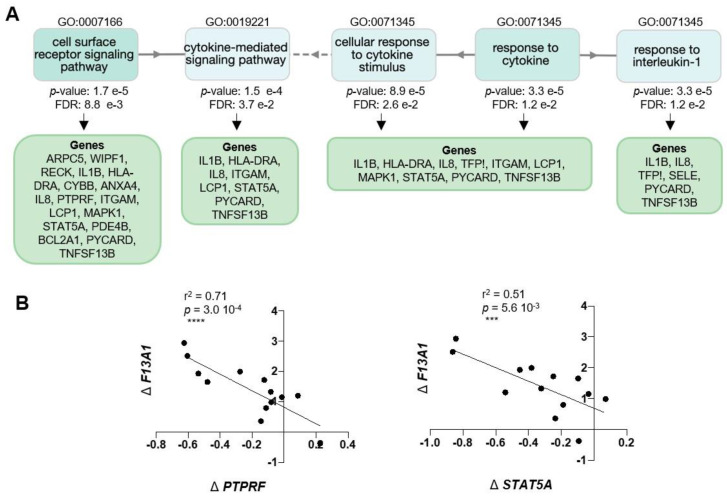
*F13A1* expression in adipocyte-enriched fraction associates with cytokine-mediated signaling pathways. (**A**) Map of GOterm nodes representing Biological Processes involved in responding to external stimuli. Gene lists for representative nodes are shown. (**B**) Linear correlation of Δ*F13A1* (Heavy–Lean twin) with differential expression Δ*gene* (Heavy–Lean twin) of selected genes from GOterms. *F13A1* levels in adipocyte-enriched fraction negatively correlate with *PTPRF*, a positive inhibitor of insulin signaling, and to *STAT5A*, a promoter of adipocyte differentiation.

**Figure 7:**
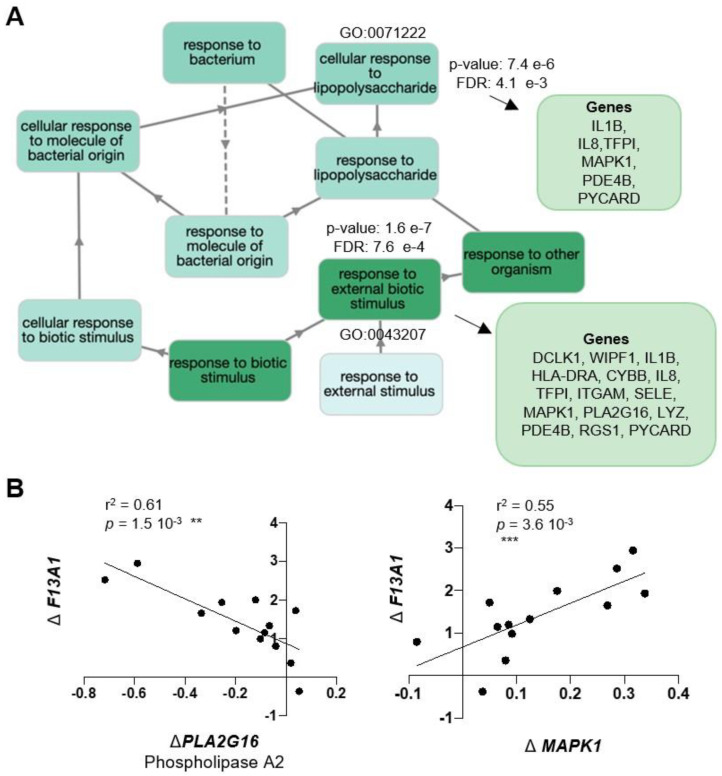
*F13A1* expression in adipocyte-enriched fraction associates with immune response to external factors and stimulus. (**A**) Map of GOterm nodes representing Biological Processes involved in responding to external stimuli. Gene lists for representative nodes are shown. (**B**) Linear correlation of Δ*F13A1* (Heavy–Lean twin) with differential expression Δ*gene* (Heavy–Lean twin) of selected genes from GOterms. *F13A1* levels in adipocyte-enriched fraction correlate negatively with *PLA2G16* (phospholipase A2), a lipolytic enzyme, and positively with expression of *MAPK1.*

Gene enrichment analysis for the 47 genes, with GOnet and Panther gene ontology tools, resulted in a total of 61 GOterms for Biological Process (BP), 13 Cellular Component (CC), and no terms for Molecular Functions. Figure 3 shows a selection [28] of the most significant, over-represented Biological Process GOterms and all 13 Cellular Component terms. The full list of GOterms is listed in Supplemental Tables S5 and S6. Further cluster analysis (Figure 4) for the GOterms using the GOnet tool revealed three clear functional clusters related to immune response, inflammation, and cytokine response and signaling. A detailed examination of each of the clusters revealed central GOterms, suggesting inflammatory response to external factors via neutrophil and leucocyte activation as well as cytokine secretion (Figure 5, Figure 6 and Figure 7). Each of the Biological Process and Cellular Component terms and some relevant genes are discussed below in more detail.

## 3. Discussion

Adipose tissue expansion through hypertrophy, where large adipocytes predominate in the expanding tissue, correlates with adipose tissue low-grade systemic inflammation and worse metabolic consequences of obesity [33,34,35,36]. The mechanisms and pathways that precede and follow the hypertrophic expansion and inflammation are numerous. Accumulating data points to sequela that involves free fatty acid (FFA)-mediated initiation of adipocyte hypertrophy, which is followed by initiation of innate immune response by the hypertrophic adipocytes [34]. Previous studies have linked two *TGM* family members, *TGM2* and *F13A1,* to adipose tissue biology and inflammation [25,26,28,29,30,37,38,39]. In this study, we have investigated the differential expression of the whole *TGM* family in adipose tissue of MZ twins that are discordant in weight (Heavy–Lean twin pairs). The MZ twins are a unique model of acquired weight gain where background involving genetic, age, sex, and early environmental influences can be controlled. The twins’ adipocity parameters and metabolism are well characterized and include adipocyte morphology, mitochondrial, fibronolytic and coagulatory, and inflammatory status [40,41,42,43,44,45,46,47]. We report that *F13A1* is the sole *TGM* family member that shows significantly altered, and in this case increased, expression in the heavier twin. Despite robust data from mouse studies linking TG2 to adipose tissue function in obesity [25,26], *TGM2* showed no altered expression between the Heavy–Lean co-twins. Further studies in larger cohorts of more severely obese individuals could shed more light into the role of TG2 in human adipose tissue pathology.

The presence of large versus small adipocytes in adipose tissue in obesity has been associated with worse metabolic outcome [18]. Our previous work linked increase in *F13A1* in adipose tissue to increased adipocyte size [30]. The aim of this current study was to further examine through which pathways *F13A1* associates to large adipocytes. This was done by screening the pool of the previously identified 182 *F13A1-* and weight-correlating genes [30] in the adipocyte-enriched fraction for: (1) genes that have been reported to associate positively or negatively to adipocyte size [17], and (2) genes that are expressed significantly more in larger adipocytes or demonstrated in the literature to regulate adipocyte size [18,31]. Here, we demonstrate tight association of *F13A1* to 47 ‘adipocyte size’ genes that via gene enrichment analysis linked to Biological Processes involving response to external stimulus, secretion, exocytosis, vesicle-mediated transport, cell surface receptor signaling, neutrophil activation, and response to interleukin 1. The GOterms for Cellular Components suggested association of *F13A1* in lysosome, secretory vesicles, plasma membrane rafts, and extracellular space. Network and cluster analysis of the GOterms link these to immune response/inflammation and cytokine response (Figure 4), which jointly suggest that *F13A1* may be involved in pro-inflammatory response from large adipocytes. In our previous study, we discussed several genes that are also among the current 47 genes and they were associated with adipocyte oxidative stress, extracellular matrix (ECM) organization and remodeling, angiogenesis, and macrophages [30]. Associations to all the above processes suggests that *F13A1* may be involved throughout in molecular events of the tissue expansion.

The adipocyte-enriched fraction used in this study was prepared from SAT biopsies by a method involving collagenase digestion followed by collecting of the released, buoyant adipocytes. This method is considered to separate adipocytes from the stromal vascular fraction that contain pre-adipocytes, mesenchymal stem cells, endothelial and vascular smooth muscle cells, and immune cells [48], however, other cell types do co-purify and contribute to the transcripts observed. Thus, the preparation enriches adipocytes, but is not void of other cell types. It has become apparent that pure adipocyte preparations require flow cytometry sorting and is more difficult than previously considered [49]. The other cell types have likely co-purified and may include macrophages whose marker *ITGAM* (Integrin αM; CD11b) is present also in this final set of 47 genes. The preparation also likely includes endothelial cells, which as suggested by the expression of genes like *SELE* (Selectin E) [30], is a product of cytokine-stimulated endothelial cells and mediates endothelial cell adhesion to leucocytes [50]. Macrophages and endothelial cells also both produce FXIII-A enzyme [3,51,52,53,54,55,56,57,58,59]. Our major future aims include detailing the cell types that are responsible for *F13A1/F13a1* production at different phases of adipose tissue expansion, which is critical to understanding where in the inflammatory sequela *F13A1/F13a1* may be involved.

Of the 47 gene transcripts that correlate with weight, *F13A1,* and adipocyte size, we have discussed four in our previous study (*CYBB*, *CTSS*, *ITMG*, and *VCAN*) [30]. Below, we will focus mostly on the new ones identified here. The most numerous GOterms for Biological Processes were associated with neutrophil and leucocyte activation (Figure 5). One of the highest *F13A1* correlating genes in this set was *PYCARD* (r^2^ = 0.89) (Figure 5B), which encodes for ASC (apoptosis-associated speck-like protein containing a CARD). *PYCARD*/ASC is made up by macrophages, where it activates inflammasome via acting as an adaptor protein between Nlrp3 and caspase-1 [60]. Caspase-1 is critical for proteolytic activation and secretion of pro-inflammatory cytokines IL1β and IL-18 [61,62]. The presence of FFAs in adipose tissue can induce activation of inflammasome [61] and its essential role in metabolic disturbances has become clear [61,63,64]. A very recent work demonstrated that the absence of these three critical inflammasome components, Nlrp3, ASC, and caspase-1, in mice protects mice from HFD-induced obesity, insulin resistance, and adipocyte hypertrophy [64]. The Heavy–Lean increase in Δ*IL1B* also shows strong linear correlation to Δ*F13A1* (r^2^ = 0.62) (Figure 5B). IL1β is one of the major pro-inflammatory cytokines whose role in metabolic dysfunction is well documented [65].

Neutrophils are the first responders of acute inflammatory reaction and are the first cells to be recruited to the site of inflammation [66,67,68,69]. They are the most populous but short-lived cell type in early inflammation and are capable of producing large quantities of cytokines and chemokines, including TNF-α, IL-1β, and IL-8, which can initiate the second wave of immune cells, such as infiltration of macrophages and lymphocytes [66,67,68,69]. The role of neutrophils in adipose tissue inflammation has also become apparent [33,70,71]. The links of *F13A1* to both neutrophil and leucocyte activation (Figure 5) supports a role in early inflammation as well as perpetuation of the pro-inflammatory milieu. The genes contributing to GOterms that link *F13A1* to neutrophil activation are, among others, *CYBB* (NADP oxidase) (Nox2), which is made by neutrophils and macrophages and responsible for generation of reactive oxygen species and oxidative stress, which in turn can activate neutrophils and neutrophil extracellular trap formation [72]. In adipose tissue, CYBB/Nox2 contributes to adipose tissue inflammation and development of insulin resistance [73]. The presence of *TNFSF13B* (TNF Superfamily Member 13b) (also known as BAFF) in the set of 47 weight and *F13A1* correlating genes also supports neutrophil links (Figure 5B). TNFSF13B/BAFF is a neutrophil-derived cytokine that supports B lymphocyte survival. B lymphocytes can tune adipose tissue inflammation via secreting cytokines and immunoglobulins, and they contribute to metabolic failure of adipose tissue [63,74,75]. BAFF is also produced by adipocytes [76]. The role of *F13A1* in inflammation in many tissues is supported by vast literature and it is expressed by macrophages and dendritic cells [51,52,77,78,79,80,81,82]. The mechanisms by which FXIII-A contributes to inflammation involve its fibrin-stabilizing function [83,84] and monocyte/macrophage adhesion [79,85]. It may also crosslink other substrates such as plasma fibronectin [28,29]. It is not known if *F13A1* is produced by neutrophils themselves.

Cluster analysis of GOterms also revealed that *F13A1* links to cytokine-mediated signaling pathways (Figure 6). Here, we demonstrate two indicators that show that *F13A1* is also negatively associated with important insulin modifiers, which is apparent from its negative correlation to *PTPRF* and *STAT5A*. The Δ*F13A1*/Δ*PTPRF* linear regression graph in Figure 6B shows that the high increase in *F13A1* correlated with a smaller increase in PTPRF. PTPRF encodes for Protein Tyrosine Phosphatase Receptor Type F (also known as LAR), which inhibits the insulin receptor activation [86,87]. *PTPRF*/LAR knockdown in adipocytes increases adipogenesis [88] and in mice, disturbances in glucose homeostasis [89]. This data interestingly links high *F13A1* to promotion to insulin signaling, which we demonstrated in 3T3-L1 adipocytes [28]; however, in mice, the *F13A1* absence improves insulin sensitivity [29]. Also, negative association to *STAT5A* (Figure 6B) (Signal Transducer and Activator of Transcription 5A), a known promoter of adipogenesis and adipocyte differentiation, supports our previous cell culture results, where FXIII-A was required for pre-adipocyte proliferation but was rapidly downregulated during adipogenesis [28]. The link to large adipocytes suggests that it is also made later during hypertrophy, however this could also mean that large adipocytes induce *F13A1* in other adipose tissue cells. Interestingly, recent advances in understanding the phenotypes and functionality of large, hypertrophic adipocytes in adipose tissue in obesity has shown that they likely function as antigen-presenting cells to active innate immunity/immune cells via MHCII and play an important role in the initiation of adipose tissue inflammation [20,90]. Mature adipocytes have also been demonstrated to express leucocyte chemo-attractants that via direct adipocyte–leucocyte interaction stimulate T-cell proliferation [91]. The correlation of *F13A1* to HLA-DRA (Figure 5B), an HLA class II histocompatibility complex component, found on the surface of antigen presenting cells, could suggest a link to large adipocytes specialized to initiation of inflammation.

The stimuli initiating *F13A1* production, cell types producing it, and cellular location/externalization and activation in adipose tissue are major questions arising from this study. Here, the increase of *F13A1* is linked to GOterms for cellular response to external stimuli and response to lipopolysaccharide (LPS), supporting the idea that *F13A1* could be induced by those factors that initiate the inflammatory reaction. The stimuli that can initiate adipose tissue inflammation are complex and numerous [33,34] and include fibrosis and alterations in extracellular matrix components and stiffness [83,92,93,94,95,96], apoptosis and necrosis of hypertrophic adipocytes [97], hypoxia from defective angiogenesis [24,98], free fatty acids (lipotoxicity) [21], and exogenous, as well as gut microbiota-derived LPS [36]. The GOterms for this cluster of Biological Processes are presented in Figure 7, and there are two genes of particular interest *PLA2G16* and *MAPK1.PLA2G16*—phospholipase 2A—a lipolytic enzyme made in hypertrophic adipocytes that produces FFAs and induces hyperlipidemia [99,100]. Coregulation between *PLA2G16* and *F13A1* could thus imply a link to FFAs. Positive association with *MAPK1* (Figure 6B) is also worthy of mention as we demonstrated that in osteoblasts, extracellular collagen type I, also produced by adipocytes, is a major regulator of FXIII-A expression and externalization [15]. *MAPK1* is also needed for lipolysis in rat and mouse adipocytes [101,102].

Our original screen contained a set of regulatory genes demonstrated to modulate adipocyte size. From the final set of genes, 46 of the 47 were large adipocyte-associated genes and only one, *REPIN1*, was a gene that has been demonstrated to regulate adipocyte size and whole-body insulin sensitivity [103]. The association between *F13A1* and *REPIN1* is negative (r = −0.77; r^2^ = 0.70; *p* = 0.0002), i.e., higher Δ*REPIN1* levels link to lower Δ*F13A1*. This and the positive association of Δ*F13A1* to ΔAdipocyte Diameter and ΔAdipocyte Volume [30] strongly suggests that majority of *F13A1* is a result of adipocyte hypertrophy-induced events in weight gain, rather than it being a gene that influences the adipocyte size. Detailed weight gain studies in mice can address when *F13a1* transcription and protein production is initiated, when and how it is activated during adipose tissue expansion, and what are the consequences of its absence or its overexpression to adipocytes and adipose tissue inflammatory cells and processes. These studies are ongoing in our laboratory.

In conclusion, our study brings further evidence to the involvement of *F13A1* in the pathological response of adipose tissue to expansion in weight gain in humans. The concepts revealed in our study suggest novel pathways linked to *F13A1* and inflammation providing many new hypotheses to be explored that will hopefully show if FXIII-A could be a target to control inflammation of adipose tissue.

## 4. Materials and Methods

### 4.1. Weight-Discordant MZ Twins

This study involved 12 rare, healthy MZ twin pairs discordant for weight (intra-pair difference in acquired excess weight ΔBMI (body mass index) ≥ 3 kg/m^2^ (males *n* = 2, females *n* = 10, aged 27, 7 ± 1.4 years)) which were identified from two population-based twin cohorts, FinnTwin16 (*n* = 2839 pairs) and FinnTwin12 (*n* = 2578 pairs) [104]. A detailed description of the twin material has been published previously [45,105]. The twins were healthy and were not on any regular medication, excluding contraceptives. Table 1 describes the clinical characteristics of the 12 twin pairs regarding weight and adipocity. This data table has been also presented in Reference [30]. All participants have given a written informed consent. The study design was approved by the Ethical Committee of the Helsinki University Central Hospital.

### 4.2. Adipose Tissue Biopsies, Adipocyte and Tissue Preparations, and RNA Extraction

Abdominal subcutaneous adipose tissue (SAT) biopsies were obtained surgically under the umbilicus under local anesthesia. Specimens were flash frozen in −80 °C liquid nitrogen. Adipocyte-enriched fraction was prepared by a previously reported protocol which involved adipose tissue digestion in 2% collagenase dissolved in DMEM/F-12 with supplements and 2% bovine serum albumin [17]. This was followed by adipocyte mRNA extraction using RNeasy Lipid Tissue Mini Kit (Qiagen, Nordic, Solletuna, Sweden) with a DNase I (Qiagen) digestion according to the manufacturer’s instructions.

### 4.3. Adipocyte Morphology

Fresh SAT biopsy specimens were digested with 2% collagenase in DMEM/F12 with supplements [17] and 2% bovine serum albumin (as above) and were used for measurement and calculation of adipocyte size in all 12 discordant twin pairs, as before [17]. After collagenase treatment, the digests were washed and maintained in the above medium and images of live adipocytes were taken with a light microscope (Zeiss, Axioplan2) using 50× magnification. A minimum of 200 cells were measured for diameter using an image processing and analysis software ImageJ (ImageJ 1.42q/Java 1. 6.0_10 32-bit), as previously described [17].

### 4.4. Affymetrix Transcriptomics

A total of 500 ng of RNA from adipocyte-enriched fraction, isolated as described above, was used for gene expression analysis on an Affymetrix U133 Plus 2.0 array (Thermo Fischer, Santa Clara, CA, USA) according to the manufacturer’s instructions and validated as described previously [45]. Hybridization, staining, and washing were performed using the Affymetrics Fluidics Station 450 and Hybridization oven 640 under standard conditions. Expression data was pre-processed with BioConductor software (open source, www.bioconductor.org) and the GC-RMA algorithm [106].

### 4.5. Transcriptome-Wide Association Study, Statistics, and Bioinformatics

Intra-pair values (ΔHeavy–Lean) for twins’ transcriptome (differential gene expression, Δ) were calculated and screened for correlation to intra-pair Δ*F13A1* expression in adipocyte-enriched fraction using R Statistical Software (https://www.r-project.org/) [107]. Significance within the *TGM* family (*TGM1-7* and *F13A1*) expression in twin pairs was calculated via analysis of variance (ANOVA) with Bonferroni post-test using Prism GraphPad software (release 8.0, San Diego, CA, USA). *p*-Values < 0.05 were considered significant. Pearson (r) correlation and r^2^ parameters were obtained for Δ*F13A1* and ΔAdipocyte Diameter, and those with r^2^ > 0.5 and associated *p*-values < 0.05 were considered for further screening. Normal distribution of *F13A1* mRNA expression values and adipocyte diameter values were assessed as before [30]. Significance of differential gene expression between the co-twins was tested with a paired T-test and associated *p*-values < 0.05 were considered significant. Further linear regression analysis was performed for selected genes with significant associations to Δ*F13A1* with Prism GraphPad software (release 8.0, San Diego, CA, USA). Here, *p*-values were considered significant as follows: * *p* < 0.05, ** *p* < 0.01, *** *p* < 0.001, **** *p* < 0.0001. Gene Ontology enrichment analysis and GOterm cluster analysis for over-represented genes from transcriptome screening was performed using Panther, GOnet, and Gorilla online tools. Significance for GOterms is represented in False Discovery Rate (FDR) adjusted *p*-values.

## Figures and Tables

**Table 1:** Clinical characteristics of the twin pairs (*n* = 12) used in this study to obtain transcriptomics data from adipose tissue and adipocyte-enriched fraction. Student’s T-test was used to calculate the differences between the twin pairs, and Wilcoxon Signed rank’s test to determine the *p*-values.

Clinical and Adipocity Parameters	Heavier Co-twin	Leaner Co-twin	*p*-Value
Height (cm)	169.3 ± 2.89	169.2 ± 3.0	0.7530
Weight (kg)	83.8 ± 4.6	67.0 ± 4.1	0.0022
BMI (kg/m^2^)	29.0 ± 0.85	23.2 ± 0.7	0.0022
Body fat (%)	41.9 ± 2.0	32.8 ±1.8	0.0029
Body fat (kg)	34.7 ± 2.3	22.0 ± 1.9	0.0022
Fat-free mass (kg)	46.6 ± 3.7	42.8 ± 2.9	0.0029
Subcutaneous fat (dm^3^)	5762.4 ± 450.6	3303.6 ± 275.4	0.0022
Intra-abdominal fat (dm^3^)	1109.8 ± 186.4	502.2 ± 91.2	0.0037
Liver fat (%)	2.4 ± 0.74	0.60 ± 0.06	0.0076
Triglycerides (mmol/L)	1.14 ± 0.07	0.96 ± 0.11	0.1823
fP-Leptin (pg/mL)	34,229.0 ± 5931.3	18,331.8 ± 4412.6	0.0037
fP-Adiponectin (ng/mL)	2844.0 ± 382.1	3520.1 ± 380.1	0.0186
Adipocyte diameter (mm)	87.9 ± 2.82	76.6 ± 2.77	0.0022
Adipocyte number	8.8e + 13 ± 5.7e + 12	8.5e + 13 ± 8.2e + 12	0.4328

BMI: body mass index, fP: fasting plasma. Data are presented as mean ± SEM.

**Table 2:** List of 47 genes showing strong and significant correlation Heavy–Lean ΔF13A1, ΔAdipocyte Diameter, and significantly altered expression between the 12 MZ co-twins. Genes are ranked based on smallest *p*-value.

		**Positive Correlation**		
**Gene Symbol**	**Gene ID**	**Gene Name**	**Pearson (r)**	***p*-Value**
*CPVL*	ENSG00000106066	Carboxypeptidase, vitellogenic-like	0.946	0.00000097
*PYCARD*	ENSG00000103490	PYD and CARD domain containing	0.941	0.00000167
*CTSS*	ENSG00000163131	Cathepsin S	0.902	0.00002544
*ITGAM*	ENSG00000169896	Integrin subunit alpha M	0.885	0.00005774
*DCLK1*	ENSG00000133083	Doublecortin-like kinase 1	0.867	0.00012405
*CYBB*	ENSG00000165168	Cytochrome b-245 beta chain	0.856	0.00018955
*SRGN*	ENSG00000122862	Serglycin	0.842	0.0003081
*TM4SF1*	ENSG00000169908	Transmembrane 4 L six family member 18	0.834	0.00039092
*PDE4B*	ENSG00000184588	Phosphodiesterase 4B	0.834	0.00039317
*TFPI*	ENSG00000003436	Tissue factor pathway inhibitor	0.83	0.00044582
*ANXA4*	ENSG00000196975	Annexin A4	0.827	0.00049387
*IL8*	ENSG00000169429	Interleukin 8	0.806	0.00087858
*VCAN*	ENSG00000038427	Versican	0.806	0.00088439
*BCL2A1*	ENSG00000140379	BCL2-related protein A1	0.802	0.00097386
*LCP1*	ENSG00000136167	Lymphocyte cytosolic protein 1/Plastin-2	0.802	0.00097956
*RECK*	ENSG00000122707	Reversion inducing cysteine-rich protein with kazal motifs	0.799	0.00105162
*ABI3BP*	ENSG00000154175	ABI family member 3 binding protein	0.795	0.00115971
*IL1B*	ENSG00000125538	Interleukin 1 beta	0.786	0.00145102
*SAMSN1*	ENSG00000155307	SAM domain, SH3 domain, and nuclear localization signals 1	0.78	0.00165797
*RGS1*	ENSG00000090104	Regulator of G protein signaling 1	0.775	0.00186205
*CCDC109B*	ENSG00000005059	Mitochondrial calcium uniporter dominant negative beta subunit	0.774	0.0018858
*NCEH1*	ENSG00000144959	Neutral cholesterol ester hydrolase 1	0.766	0.0022702
*ARPC5*	ENSG00000162704	Actin-related protein 2/3 complex subunit 5	0.764	0.00235294
*LYZ*	ENSG00000090382	Lysozyme	0.762	0.00247521
*MAPK1*	ENSG00000100030	Mitogen-activated protein kinase 1	0.743	0.00359262
*HLA-DRA*	ENSG00000204287	Major histocompatibility complex, class II, DR alpha	0.743	0.00361775
*ARHGAP18*	ENSG00000146376	Rho GTPase activating protein 18	0.743	0.00364017
*TNFSF13B*	ENSG00000102524	TNF superfamily member 13b/B cell activation factor	0.739	0.00393293
*WIPF1*	ENSG00000115935	WAS/WASL interacting protein family, member 1	0.728	0.00481724
*CNKSR3*	ENSG00000153721	CNKSR family member 3	0.725	0.00503039
*MGP*	ENSG00000111341	Matrix Gla protein	0.72	0.00555039
*EVI2A*	ENSG00000126860	Ecotropic viral integration site 2A	0.717	0.00579394
*NPTN*	ENSG00000156642	Neuroplastin	0.715	0.00596749
*SELE*	ENSG00000007908	Selectin-E	0.778	0.00174649
*LPCAT1*	ENSG00000153395	Lysophosphatidylcholine acyltransferase 1	0.732	0.00445358
		**Negative Correlation**		
**Gene Symbol**	**Gene ID**	**Gene Name**	**Pearson (r)**	***p*-Value**
*SCRN2*	ENSG00000141295	Secernin 2	–0.876	0.000086
*PTPRF*	ENSG00000142949	Protein tyrosine phosphatase receptor type F	–0.84	0.00033
*EMC3*	ENSG00000125037	ER membrane protein complex subunit 3	–0.816	0.000673
*ABCD1*	ENSG00000101986	ATP binding cassette subfamily D member 1	–0.793	0.001229
*PLA2G16*	ENSG00000176485	Phospholipase A and acyltransferase 3	–0.784	0.001527
*NFRKB*	ENSG00000170322	Nuclear factor related to kappaB binding protein	–0.74	0.00386
*HEPACAM*	ENSG00000165478	Hepatic and glial cell adhesion molecule	–0.734	0.00426
*RBP1*	ENSG00000114115	Retinol binding protein 1, cellular	–0.725	0.00501
*STAT5A*	ENSG00000126561	Signal transducer and activator of transcription 5A	–0.72	0.00556
*TUSC5*	ENSG00000184811	Tumor suppressor candidate 5/Trafficking regulator of GLUT4	–0.717	0.005843
*COX5A*	ENSG00000178741	Cytochrome c oxidase subunit Va	–0.712	0.006346
*REPIN1*	ENSG00000214022	Replication initiator 1	–0.772	0.001987

## Supplementary Materials

The following are available online at https://www.mdpi.com/1422-0067/21/21/8289/s1. References [17,31,108,109,110,111,112,113,114,115,116,117,118,119,120] are cited in the supplementary materials.
