# Supplementary material for: Transglutaminases and Obesity in Humans: Association of F13A1 to Adipocyte Hypertrophy and Adipose Tissue Immune Response
Source: Int J Mol Sci. 2020 Nov 5;21(21):8289. doi: 10.3390/ijms21218289 (PMC7663854; doi:10.3390/ijms21218289)
Supplement: Supplementary file 1 [file ijms-21-08289-s001.zip › ijms-934223 sup/ijms-934223 Sup S1-4.docx]

**Supplemental Table S1** Genes reported to correlate positively to adipocyte size in monozygotic twins discordant in weight.

| **GENE** | **ID** | **REFERENCE** |
| --- | --- | --- |
| *FLNA* | ENSG00000196924 | [17] |
| *BAD* | ENSG00000002330 | [17] |
| *MAGED1* | ENSG00000179222 | [17] |
| *ZMAT3* | ENSG00000172667 | [17] |
| *ATR* | ENSG00000175054 | [17] |
| *CCND1* | ENSG00000110092 | [17] |
| *CCND2* | ENSG00000118971 | [17] |
| *PLXNA1* | ENSG00000114554 | [17] |
| *RUVBL1* | ENSG00000175792 | [17] |
| *POLE4* | ENSG00000115350 | [17] |
| *CYBA* | ENSG00000051523 | [17] |
| *TK2* | ENSG00000166548 | [17] |
| *PAFAH2* | ENSG00000158006 | [17] |
| *CBR1* | ENSG00000159228 | [17] |
| *AKR1C3* | ENSG00000196139 | [17] |
| *PPAP2A* | ENSG00000067113 | [17] |
| *PLA2G4A* | ENSG00000116711 | [17] |
| *MSN* | ENSG00000147065 | [17] |
| *NHEDC2* | ENSG00000164038 | [17] |
| *RP11-877E17.2* | ENSG00000246695 | [17] |
| *KIF3B* | ENSG00000101350 | [17] |
| *NME5* | ENSG00000112981 | [17] |
| *IFT20* | ENSG00000109083 | [17] |
| *MIF* | ENSG00000240972 | [17] |
| *SLC24A3* | ENSG00000185052 | [17] |
| *C15orf59* | ENSG00000205363 | [17] |
| *CD248* | ENSG00000174807 | [17] |
| *SLC46A3* | ENSG00000139508 | [17] |
| *XPO6* | ENSG00000169180 | [17] |
| *FAT1* | ENSG00000083857 | [17] |
| *GNG2* | ENSG00000186469 | [17] |
| *LPCAT1* | ENSG00000153395 | [17] |
| *TCTA* | ENSG00000145022 | [17] |
| *CLTB* | ENSG00000175416 | [17] |
| *SPTAN1* | ENSG00000197694 | [17] |
| *CYBASC3* | ENSG00000162144 | [17] |

**Supplemental Table S2** Genes reported to correlate negatively to adipocyte size in monozygotic twins discordant in weight.

| **GENE** | **ID** | **REFERENCE** |
| --- | --- | --- |
| *RNF125* | ENSG00000101695 | [17] |
| *SMAD4* | ENSG00000141646 | [17] |
| *PKN2* | ENSG00000065243 | [17] |
| *CCNG1* | ENSG00000113328 | [17] |
| *MAP3K5* | ENSG00000197442 | [17] |
| *POLR1D* | ENSG00000186184 | [17] |
| *CLDN3* | ENSG00000165215 | [17] |
| *AGPAT9* | ENSG00000138678 | [17] |
| *AASS* | ENSG00000008311 | [17] |
| *ACSS3* | ENSG00000111058 | [17] |
| *HADHB* | ENSG00000138029 | [17] |
| *BDH1* | ENSG00000161267 | [17] |
| *NPEPPS* | ENSG00000141279 | [17] |
| *GLYCTK* | ENSG00000168237 | [17] |
| *PKP2* | ENSG00000057294 | [17] |
| *AZGP1* | ENSG00000160862 | [17] |
| *CIDEA* | ENSG00000176194 | [17] |
| *FAM184A* | ENSG00000111879 | [17] |
| *NUP98* | ENSG00000110713 | [17] |
| *WHSC2* | ENSG000001 85049 | [17] |
| *FAM161A* | ENSG00000170264 | [17] |
| *SLC27A2* | ENSG00000140284 | [17] |
| *ZFAND1* | ENSG00000104231 | [17] |
| *MACROD1* | ENSG00000133315 | [17] |
| *PPARA* | ENSG00000186951 | [17] |
| *GPD1L* | ENSG00000152642 | [17] |
| *BBC3* | ENSG00000105327 | [17] |
| *CYP3A7* | ENSG00000160870 | [17] |
| *TOE1* | ENSG00000132773 | [17] |
| *EIF1B* | ENSG00000114784 | [17] |
| *ADH1B* | ENSG00000196616 | [17] |
| *FDFT1* | ENSG00000079459 | [17] |

**Supplemental Table S3** Genes reported to show higher expression in large versus small adipocytes.

| **GENE** | **ID** | **REFERENCE** |
| --- | --- | --- |
| *SELE* | ENSG00000007908 | [31] |
| *SPARCL1* | ENSG00000152583 | [31] |
| *TM4SF1* | ENSG00000169908 | [31] |
| *DCN* | ENSG00000011465 | [31] |
| *IL8* | ENSG00000169429 | [31] |
| *PALLD* | ENSG00000129116 | [17,31] |
| *SAA2* | ENSG00000134339 | [31] |
| *CLEC3B* | ENSG00000163815 | [31] |
| *C1QR1* | ENSG00000125810 | [31] |
| *COL1A1* | ENSG00000108821 | [31] |
| *CXCL2* | ENSG00000081041 | [31] |
| *COL1A2* | ENSG00000164692 | [31] |
| *FLJ14054* | ENSG00000113389 | [31] |
| *AQP1* | ENSG00000240583 | [31] |

**Supplemental Table S4** Genes reported to regulate adipocyte size.

| **GENE** | **ID** | **REFERENCE** |
| --- | --- | --- |
| *LRRC8A* | ENSG00000136802 | [108] |
| *CIDEA* | ENSG00000176194 | [109] |
| *NUP85* | ENSG00000125450 | [110] |
| *HID1* | ENSG00000167861 | [110] |
| *CASKIN2* | ENSG00000177303 | [110] |
| *FAM13A* | ENSG00000138640 | [111] |
| *POM121C* | ENSG00000272391 | [111] |
| *SNTB2* | ENSG00000168807 | [112] |
| *TCF7L2* | ENSG00000148737 | [113] |
| *DLK1* | ENSG00000185559 | [114] |
| *ZFP423* | ENSG00000102935 | [115] |
| *APOE* | ENSG00000130203 | [116] |
| *BMP4* | ENSG00000125378 | [117] |
| *EBF1* | ENSG00000164330 | [117] |
| *REPIN1* | ENSG00000214022 | [118] |
| *MEST* | ENSG00000106484 | [119] |
| *AGT2R* | ENSG00000180772 | [120] |
